# Intraoperative landmarking errors and their avoidance in robotic‐assisted total knee arthroplasty using the VELYS system

**DOI:** 10.1002/jeo2.70902

**Published:** 2026-09-07

**Authors:** Alexander Aichmair, Heiko Graichen, Nimrod Snir, Benjamin V. Bloch, Ugonna N. Ihekweazu, Jochen G. Hofstaetter, Michael T. Hirschmann, Pieter‐Jan Vandekerckhove

**Affiliations:** ^1^ Department of Arthroplasty Orthopaedic Hospital Speising Vienna Austria; ^2^ Orthopaedic Hospital Speising Michael Ogon Laboratory for Orthopaedic Research Vienna Austria; ^3^ Department of Personalised Orthopaedics (PersO) Privatklinik Siloah Berne Switzerland; ^4^ Division of Orthopedics Tel Aviv Sourasky Medical Center Tel Aviv‐Yafo Israel; ^5^ Nottingham Elective Orthopaedic Service Nottingham University Hospitals NHS Trust Nottingham UK; ^6^ Fondren Orthopedic Group Fondren Orthopedic Research Institute Houston Texas USA; ^7^ University Department of Orthopaedic Surgery and Traumatology Kantonsspital Baselland Bruderholz Switzerland; ^8^ Department of Orthopaedic Surgery and Traumatology Sint‐Jan Hospital Bruges Belgium

**Keywords:** accuracy, landmarking errors, robotic, technical errors, total knee arthroplasty

## Abstract

While robotic‐assisted total knee arthroplasty (rTKA) has been proven to achieve highly precise and reproducible radiographic results, its accuracy remains highly dependent on correct intraoperative landmark acquisition. This technical guide aims to provide practical guidance for intraoperative recognition of landmarking and gap balance assessment errors during rTKA using the image‐free VELYS^TM^ system. Based on the authors' clinical experience with this system, the most common pitfalls as well as their intraoperative recognition are highlighted. Special focus is set on pin placement, bone reference checkpoints and landmark acquisition, with practical intraoperative cues that allow surgeons to detect these errors intraoperatively before bone resections are performed. The present article should support early adopters of the VELYS^TM^ system, with the aim of preventing precision inaccuracies.

**Level of Evidence:**

Level V.

**Abbreviations**
3D: three‐dimensional
MPTA: medial proximal tibial angle
PS: posterior stabilized
ROM: range of motion
rTKA: robotic‐assisted total knee arthroplasty
TKA: total knee arthroplasty

## INTRODUCTION

Total knee arthroplasty (TKA) is a highly successful procedure for the treatment of end‐stage osteoarthritis of the knee [19]. While mechanical alignment has been considered the gold standard for decades [13, 18], personalized alignment strategies have been established with the aim of improving postoperative patient satisfaction and outcomes [1, 17, 18]. Reproducible landmarking and gap measurements are the basis for detecting the individual bony [9, 10, 15] and laxity phenotype [6, 7, 8], the basis for personalized alignment. Robotic‐assisted total knee arthroplasty (rTKA) has been introduced to enable accurate intraoperative planning, real‐time feedback and precise execution of bone resections. Several robotic platforms have demonstrated improved alignment accuracy and reduced outliers compared with conventional and navigated techniques [2, 3].

The VELYS™ robotic‐assisted system is a recently introduced image‐free robotic platform that has increasingly gained clinical adoption due to multiple factors, such as promising accuracy in component positioning and limb alignment [2]. Despite technological advances, the accuracy remains highly dependent on the correct intraoperative acquisition of bony landmarks and gap measurements. Landmarking errors as well as laxity measurement errors may propagate through the planning and execution phases, potentially resulting in unintended deviations between the planned and achieved component alignment. While overall system accuracy and efficacy have been the focus of previous studies [2, 3, 11, 12], the specific impact of landmarking inaccuracies and laxity measurement errors and their intraoperative recognition have received limited attention in the current literature.

This paper aims to support early adopters of the VELYS™ system by facilitating intraoperative assessment of (1) correct bony landmark acquisition and (2) accurate and reproducible gap measurement. Common pitfalls of both registration parts are highlighted, and practical strategies to identify and mitigate these errors are provided to help prevent alignment inaccuracies.

## PIN PLACEMENT

The following recommendations reflect the authors' individual experience with the VELYS^TM^ robotic system rather than the results of biomechanical analyses. The reported specific angulation recommendations as well as figures should be understood as practical guidance rather than evidence‐based boundaries.

To mount the array clamps, two pins are inserted into both the tibia and femur, ensuring they are parallel to each other. The following recommendations are intended for intraincisional pin placement; however, extraincisional options are also possible. In TKA, placement of the pins within the incision is safe, avoiding the risk of diaphyseal fractures and pin site wound complications or infections [4].

### Femoral pin placement

**Figure 1:**
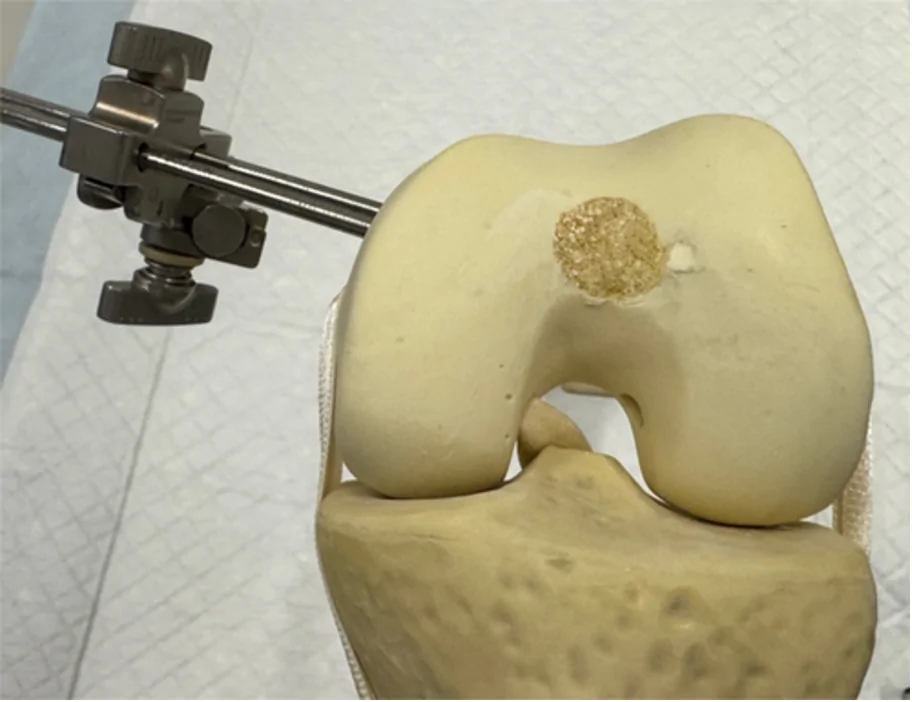
Axial orientation of approximately 20°–30° relative to the transepicondylar axis of the femur is recommended to avoid potential conflict between the robot and the array fixation.

When positioning the femoral pins for mounting the array clamps, the authors recommend attention to the correct position of the pins to avoid conflicts between the robotic arm and pins during the surgery. According to the authors, a favourable trajectory is (a) parallel to the femoral joint line, (b) in the anteroposterior centre of the bone and (c) approximately 1–2 cm proximal to the femoral epicondyle in the metaphysis. For the axial orientation of the pins, an angulation of approximately 20°–30° relative to the transepicondylar axis is used by the authors, with the two pins in line with the femoral diaphysis (Figure 1). This allows the surgeon to easily orient the arrays for optimal visibility, without risking a conflict with the saw during the surgical steps of femoral cuts. The arrow marking on the clamp should point towards the camera; however, if impingement between clamp basis and clamp occurs in this position, it can easily be turned 180°. Then the arrow is pointing away from the camera; however, all measurements remain unaffected.

**Figure 2:**
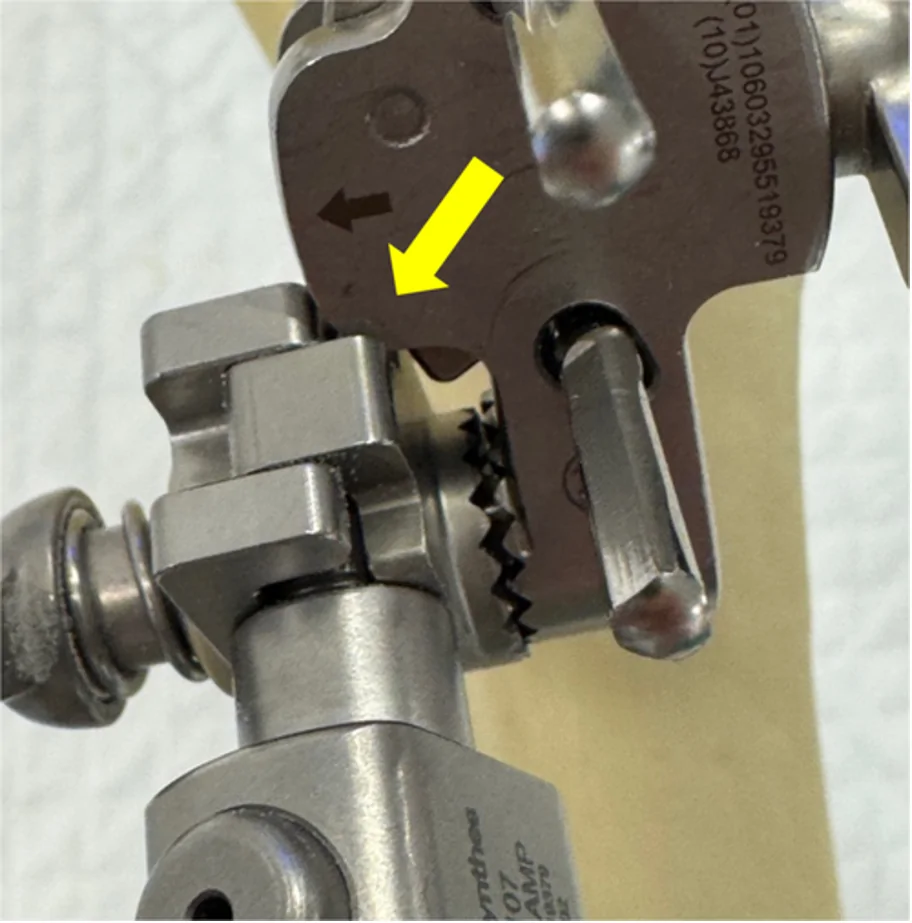
In case of malorientation of the pins for array clamp fixation, a conflict might result between the clamp base and clamp arm (yellow arrow). In case of such an impingement, the teeth of the clamp mechanism will not be properly engaged (‘tooth‐on‐tooth’ pattern). Due to an insufficient closure mechanism, the array position may shift during surgery, which could introduce potential errors.

**Figure 3:**
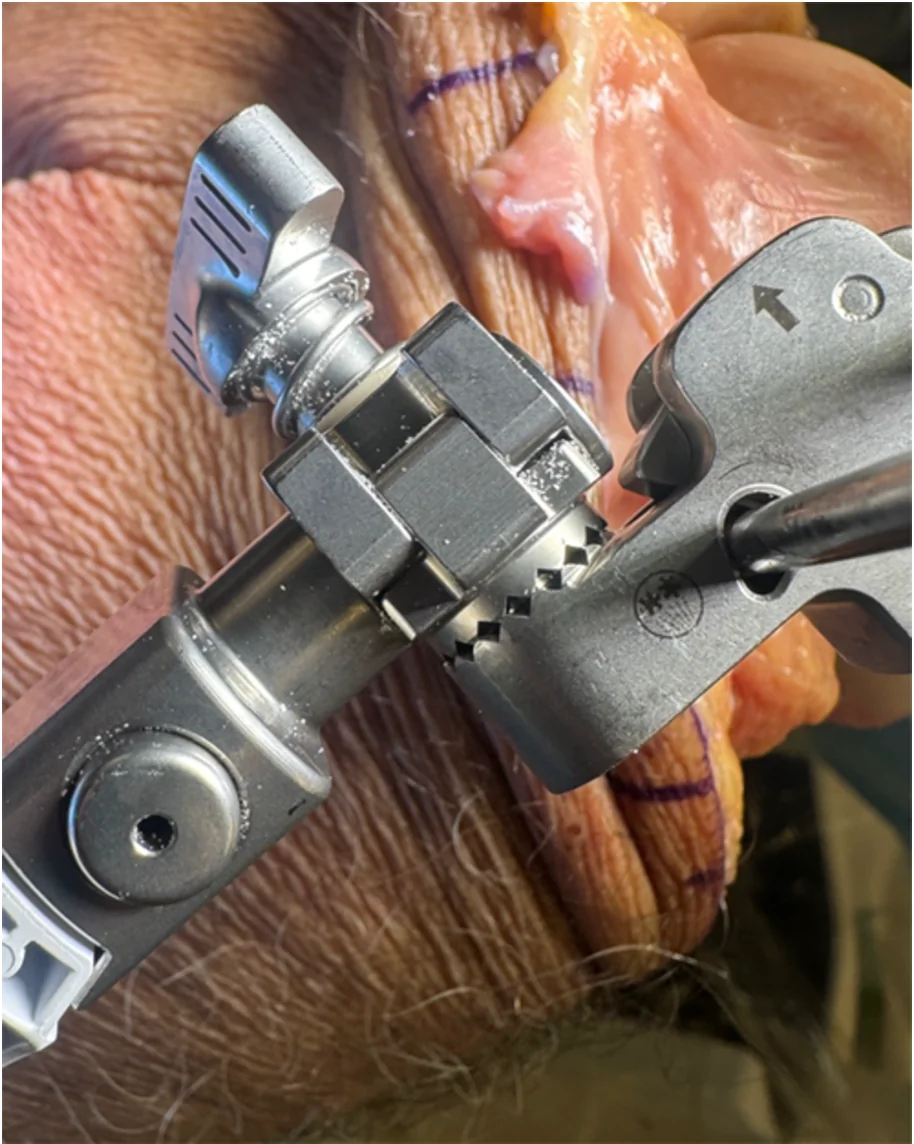
Tooth‐on‐tooth fixation is also possible even in the absence of impingement between clamp base and arm. Surgeons should carefully check that the clamp is fully engaged.

If femoral pins are inserted at an angulation towards the femoral joint line, they may interfere with the array clamp. As illustrated in Figures 2 and 3, if there is impingement between the arm and base of the clamp, the teeth of the clamp mechanism will not be properly engaged. If this ‘tooth‐on‐tooth’ pattern is not noticed during landmarking, the array position may shift during surgery, which could introduce potential errors. Should this occur after bone cuts have already been performed, converting to manual instrumentation is strongly recommended by the authors.

**Figure 4:**
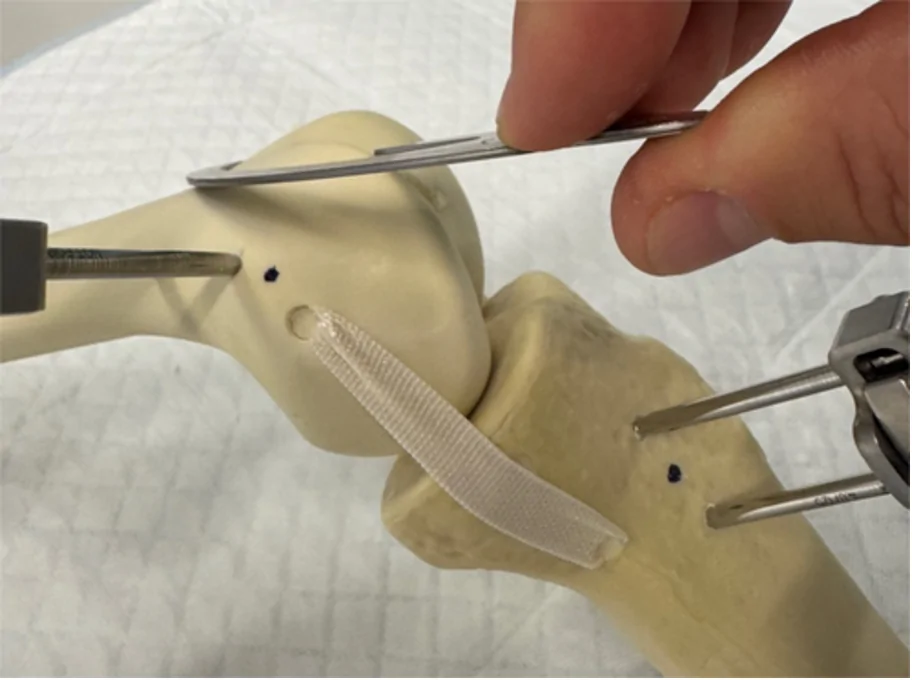
An angel wing can be used to double‐check correct pin placement to avoid conflict between the projected anterior femoral bone cut plane with the pins and bone validation points.

If the pins are placed too anteriorly in the anteroposterior dimension, the pin position may be in conflict with the anterior femoral cut. The angel wing can be used intraoperatively to double‐check (Figure 4). If the pins are placed too posteriorly, there is a theoretical risk of insufficient pin fixation and neurovascular injury. Finally, when using a posterior‐stabilized (PS) femoral component, the recommended femoral pin trajectory should avoid any pin conflict with the box cut for the PS femur.

### Tibial pin placement

**Figure 5:**
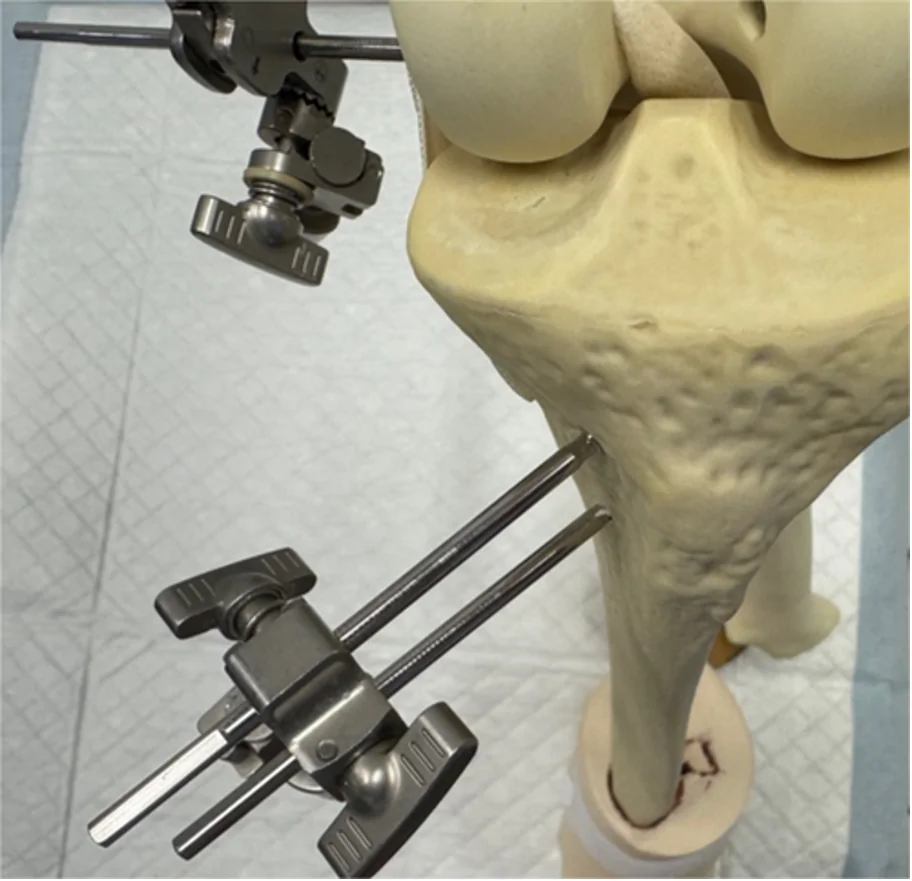
A trajectory of approximately 30°–40° relative to the tibia, perpendicular to the medial metaphyseal tibial surface, is optimal.

As illustrated in Figure 5, according to the authors' experience, pin placement in 30°–40° relative to the tibial plateau orientation allows enough space for saw handling. The authors place the tibial pins approximately in the middle of the medial metaphyseal tibial surface rather than too far anteriorly, to avoid injury to the tibial tuberosity. As a practical rule of thumb derived from the authors' experience, pin placement 2–3 cm distal to the tibial joint line is generally sufficient, without enlarging the skin incision too far distally. This is particularly true if the final balance measurements are to be obtained using the trial tibial component. If the surgeon wants to take the final balance measurement with the definitive implant in place, however, the pins should be placed at least 3.5–4 cm distal to the tibial plateau surface to allow for the implant keel.

**Figure 6:**
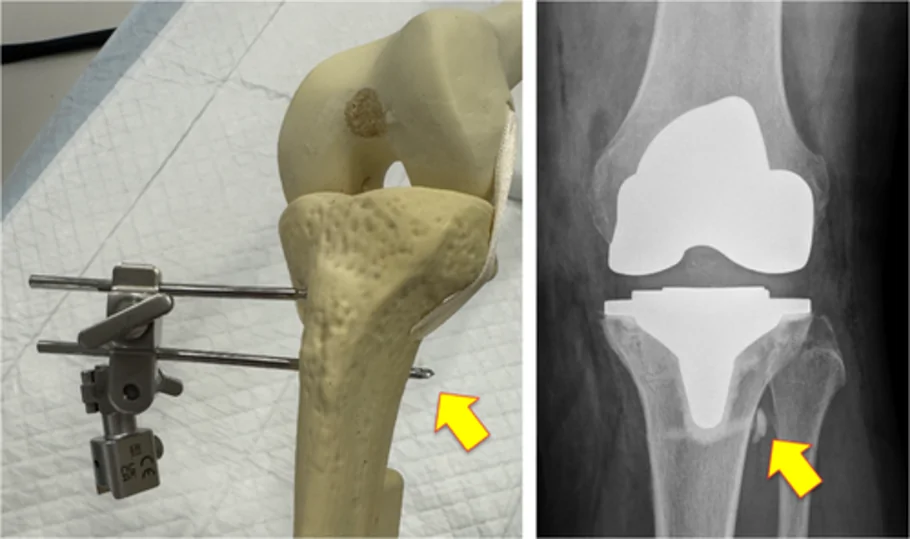
Bicortical pin perforation might result in neurovascular injury and cement extravasation (yellow arrow).

As for femoral pin placement, the authors place the tibial pins parallel to the tibial joint line to reduce the risk of an impingement between the clamp base and clamp arm and tooth‐on‐tooth clamp engagement. Especially in patients with smaller tibiae, care should be taken not to drill the pins too deep into the bone, due to the shape of the tibial metaphysis. This may potentially result in neurovascular or soft tissue damage or cement extravasation posteriorly (Figure 6). In patients with poor bone quality, bicortical pin placement may be required to achieve solid fixation. Careful drilling and correct placement are therefore recommended to avoid potential problems.

### Camera visibility

The camera must maintain a clear line of sight to the arrays during all motion and gap measurements. The authors recommend positioning femoral and tibial arrays in an ‘open book’ orientation facing the camera for optimal visualization. Minor adjustments, such as femoral or tibial internal rotation, may improve visibility of the arrays. The visibility of the arrays can be checked throughout the range of motion (ROM) before any landmark registration is performed, as described in the manufacturer's user guide [20].

## BONE REFERENCE CHECKPOINTS

**Figure 7:**
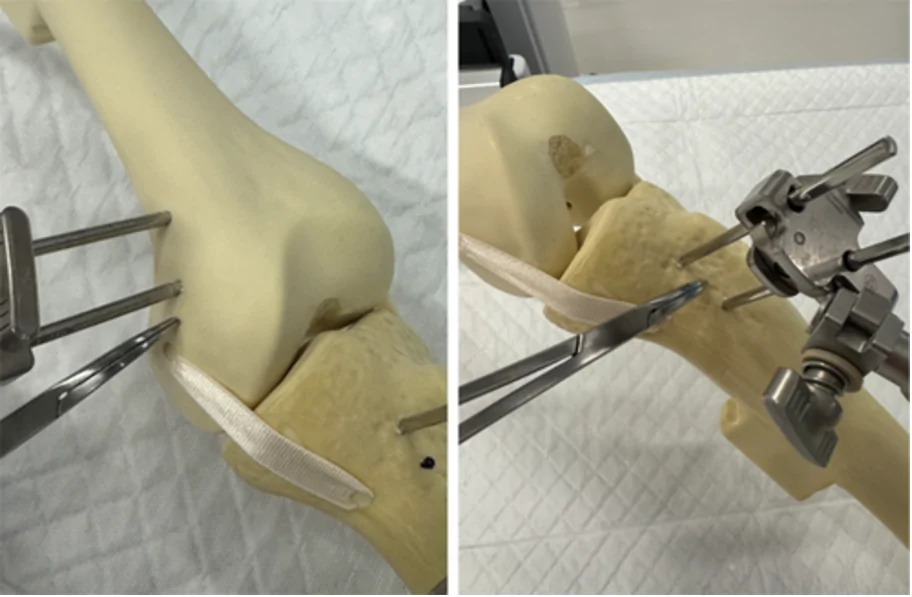
A clamp can be used to slightly impress the cortical bone for reproducible definition of validation point location.

In robotic arm‐assisted knee arthroplasty, a femoral and tibial validation point on the bone surface is used to check for potential movement of the pins during surgery. If significant array movement remains undetected, bone cuts would be performed in the wrong plane with potentially disastrous consequences for gap balancing and bony alignment. A clamp or an additional pin can be used to slightly indent the cortical bone, without perforation into the spongiosa [2, 3, 11, 12] (Figure 7). In osteoporotic bone, especially in the femoral metaphysis, one needs to be cautious not to make the indentation too deep, as this might result in an unreproducible assessment of validation points. Once the validation point has been defined, further marking with a sterile pen can be performed to rapidly recover the point during the surgery. Alternatively, small cortical screws can be used for referencing.

**Figure 8:**
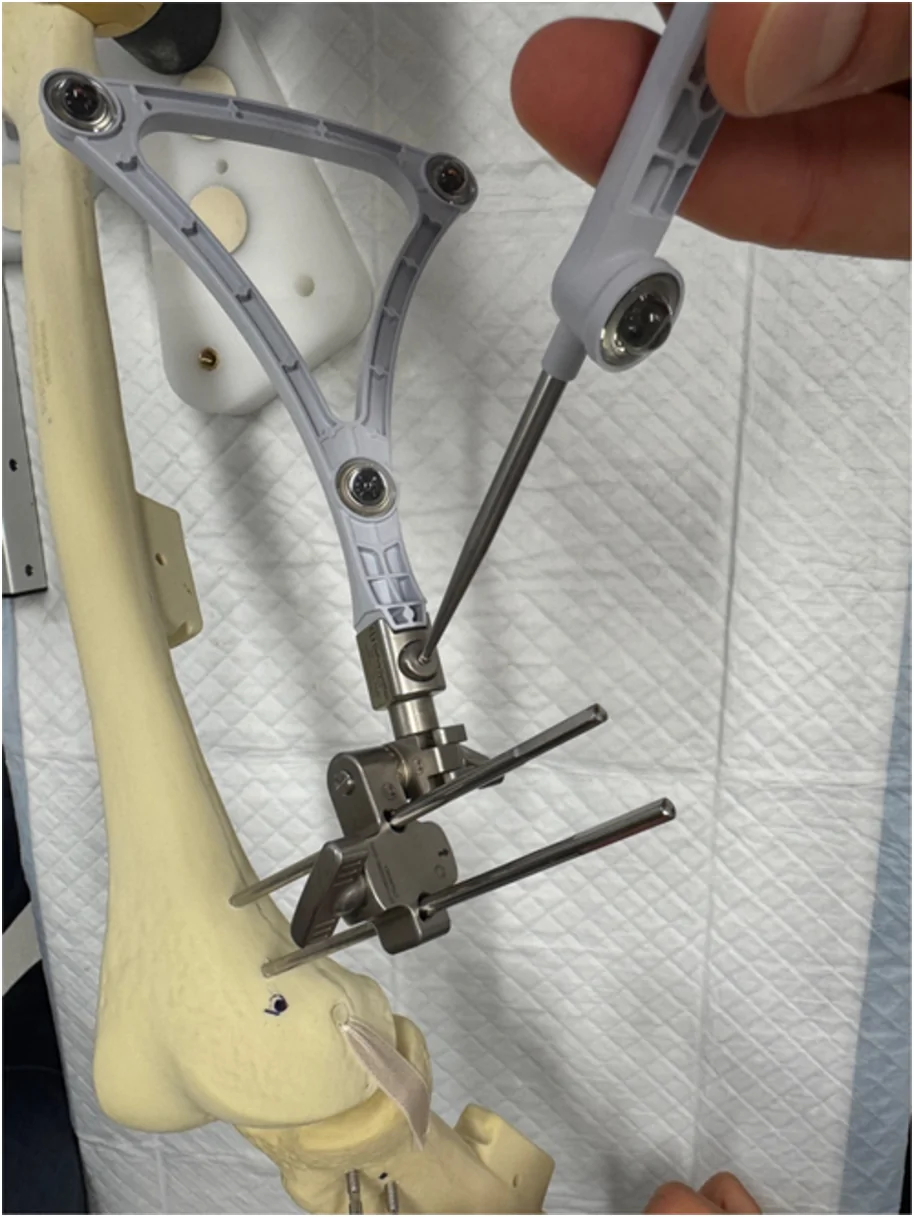
There are reports that some surgeons define the validation points on the array clamp itself. Although the validation point can be located quickly; however, it will also identify the array position as acceptable, even if the position of the pins has significantly changed. This technique is not recommended.

In the authors' experience, the cortical validation points on both the femur and tibia must be sufficiently far from the pins to reliably identify any array movement. This allows the surgeon to confirm the arrays have not moved prior to resections. It has been reported that some surgeons create the validation points on the array clamp itself, or at the pin entry point into the bone in direct contact with the pin. It is strongly advised by the authors to avoid this technique, as even a large array movement will remain undetected, as the system will not identify a change in distance between the array and the checkpoint (Figure 8).

The bony reference points should be checked prior to tibial and femoral bone cuts, with the knee in the same position as when the initial checkpoints were acquired.

## LANDMARK REGISTRATION

Landmark registration involves identifying the bony anatomical points as well as the axes of femur and tibia, which are the basis for creating the bone model and analysing gap data. Accurately registering these landmarks and bony surfaces, along with performing gap analysis, is important for precise and repeatable results. Certain factors must be carefully managed to prevent errors in landmarking. Most mistakes become apparent during surgery, so understanding how these errors can affect overall alignment enables surgeons to achieve consistent outcomes. The following recommendations are based on the authors' clinical experience with this specific robotic system and should therefore be interpreted as practical guidance rather than evidence‐based facts.

### Preparation

To obtain an accurate balancing value for the ligamentous tension, the authors recommend removing the anterior cruciate ligament, the medial and lateral meniscus, as well as any osteophytes as completely as possible before starting landmark registration. The release of these structures at a later time point during surgery may influence gap measurements, potentially impacting the accuracy of bone cuts as well.

### Hip centre of rotation

The mechanical axis of the femur is defined as the line between the hip centre of rotation and the distal femoral knee centre. During assessment of the hip centre of rotation, the authors recommend slightly flexing both hip and knee joints in order to achieve 40°–90° of hip flexion and circumducting the hip in a smooth and gentle rotation. The arrays must be visible to the camera during the entire registration procedure. It is important to avoid tilting of the pelvis during this step, as well as contact of the femoral array with the contralateral leg of the patient. Furthermore, the camera must remain locked and stable during the registration process. If the hip joint has limited ROM, then the pelvis can be stabilized by the surgical assistant, or hip circumduction can be reduced. In the authors' view, ankylosed hips are a contraindication for the use of robotic systems, as the femoral head centre cannot be accurately calculated.

### Distal femoral knee centre

The distal femoral knee centre is defined by a single point registration at the intercondylar notch apex. It is important to note that this point is significantly more posterior in the axial plane of the femur compared to the entry point used in conventional manual instrumentation techniques. Because only a single point on the distal femur is used to define the mechanical axis, the accurate definition of this point is particularly important. A falsely defined mechanical axis of the femur might also lead to a difference between the intraoperative planning and the postoperative outcome. This is particularly relevant for sagittal parameters, with less influence in the coronal plane. A medio‐laterally misplaced point will only have a minimal effect on a potential varus/valgus error, while sagittal misplacement may have a big effect on the sagittal axis.

**Figure 9:**
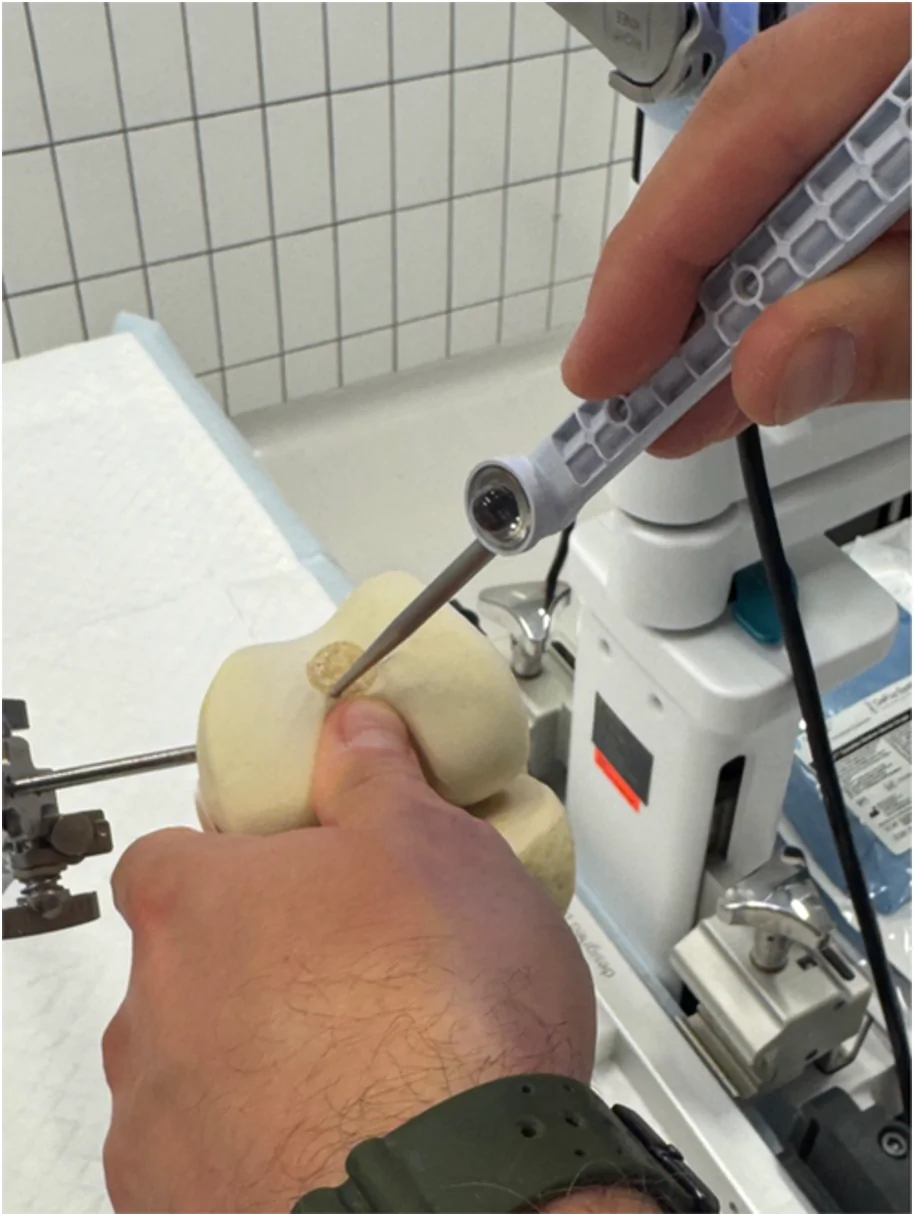
In order to correctly register the femur knee centre, the surgeon can put the thumb into the intercondylar notch after osteophyte removal and to register the landmark approximately 2 mm anterior to the tip of the thumb.

If the femoral knee centre is referenced too far anteriorly, the mechanical axis also gets shifted too far anteriorly. This error will reduce the resulting femoral flexion. The likelihood of a distal femoral knee centre being defined excessively posteriorly is, in the authors' experience minimal, given the anatomical characteristics of the intercondylar notch. However, if this does occur, the femoral flexion will be falsely increased. Osteophytes should be removed from the intercondylar notch first. One technique is to place the thumb within the notch and to register the femur centre point 2 mm anterior to the thumb (Figure 9).

**Figure 10:**
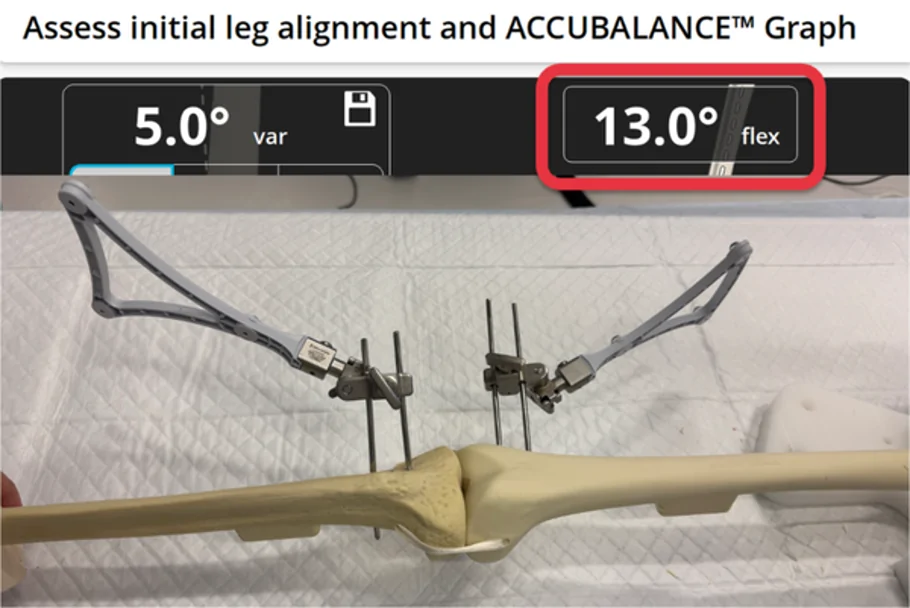
Errors in the definition of mechanical axes of femur and tibia might result in a calculated fixed flexion deformity (highlighted in red) by the robot, even though full extension is achieved clinically.

If the mechanical axis is shifted anteriorly, especially in combination with an anteriorly shifted tibia axis, the robot may wrongly calculate a fixed flexion deformity despite full extension being clinically achieved (Figure 10). Similarly, a posteriorly shifted mechanical axis may result in a falsely calculated hyperextension.

### Medial and lateral malleolus

The tibia mechanical axis is defined as the line between the single point registration of the proximal tibia knee centre and the midpoint of the malleoli (defined as the midpoint between the single points of the most prominent aspect of the medial and lateral malleoli). As only these three points are used to define this axis, it is important that attention is paid to accurate landmark registration before surgery. As described for the femoral axis, errors in the tibial axis may also negatively affect three‐dimensional (3D) knee alignment.

A possible mistake is palpating the malleoli during registration and placing the pointer too posteriorly. This may result in an overly anterior tibial axis. This error could also be exacerbated by additionally registering the tibia knee centre too far anteriorly. In both cases, an incorrect tibia slope will be calculated, and the assumption of the mechanical tibia axis might lead to a falsely calculated fixed flexion deformity as described above.

### Tibia knee centre

Defined as the intersection between the mid‐coronal and mid‐sagittal lines of the tibial plateau, the tibia knee centre is recommended by the authors to be registered at the posterior aspect of the anterior cruciate ligament footprint on the tibia. Care should be taken to avoid incorrect landmark definition, as it can adversely affect tibia slope.

### Tibia sagittal axis

**Figure 11:**
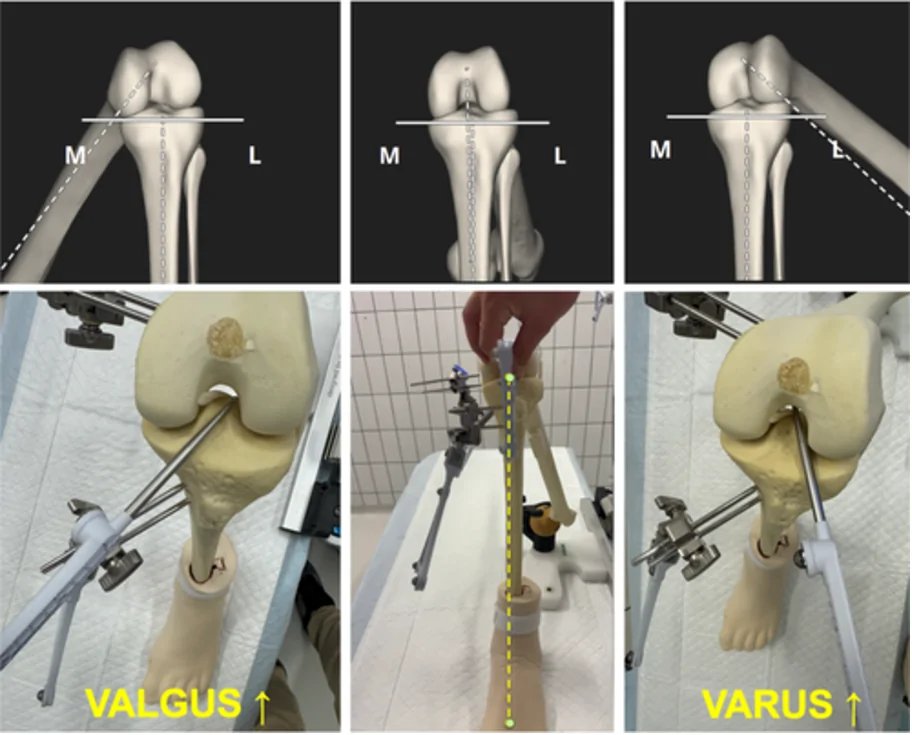
The illustration shows a correctly defined tibial sagittal axis (centre), with examples of excessive internal malorientation (left) and excessive external malorientation (right) of the pointer.

Correct definition of the neutral tibial sagittal axis through the centre of the tibia is important, as increased external or internal orientation of the pointer during this step may lead to an oblique resulting slope. On the visualization of the 3D model of the knee during ligament tension assessment, an incorrect landmarking of that axis can easily be recognized. If the sagittal axis is falsely internally rotated, the femoral head will appear medial to the tibia on the 3D visualization screen during flexion and vice versa. Ideally, in the absence of any rotational pathologies, the mechanical axes of tibia and femur should be in line when the knee is flexed (Figure 11).

If the pointer is placed in excessive internal rotation during acquisition, an oblique slope will lead to a valgus orientation of the tibia cut in a neutrally planned tibia. Excessive external rotation of the pointer will lead to a varus orientation of the tibia cut in a neutrally planned tibia.

**Figure 12:**
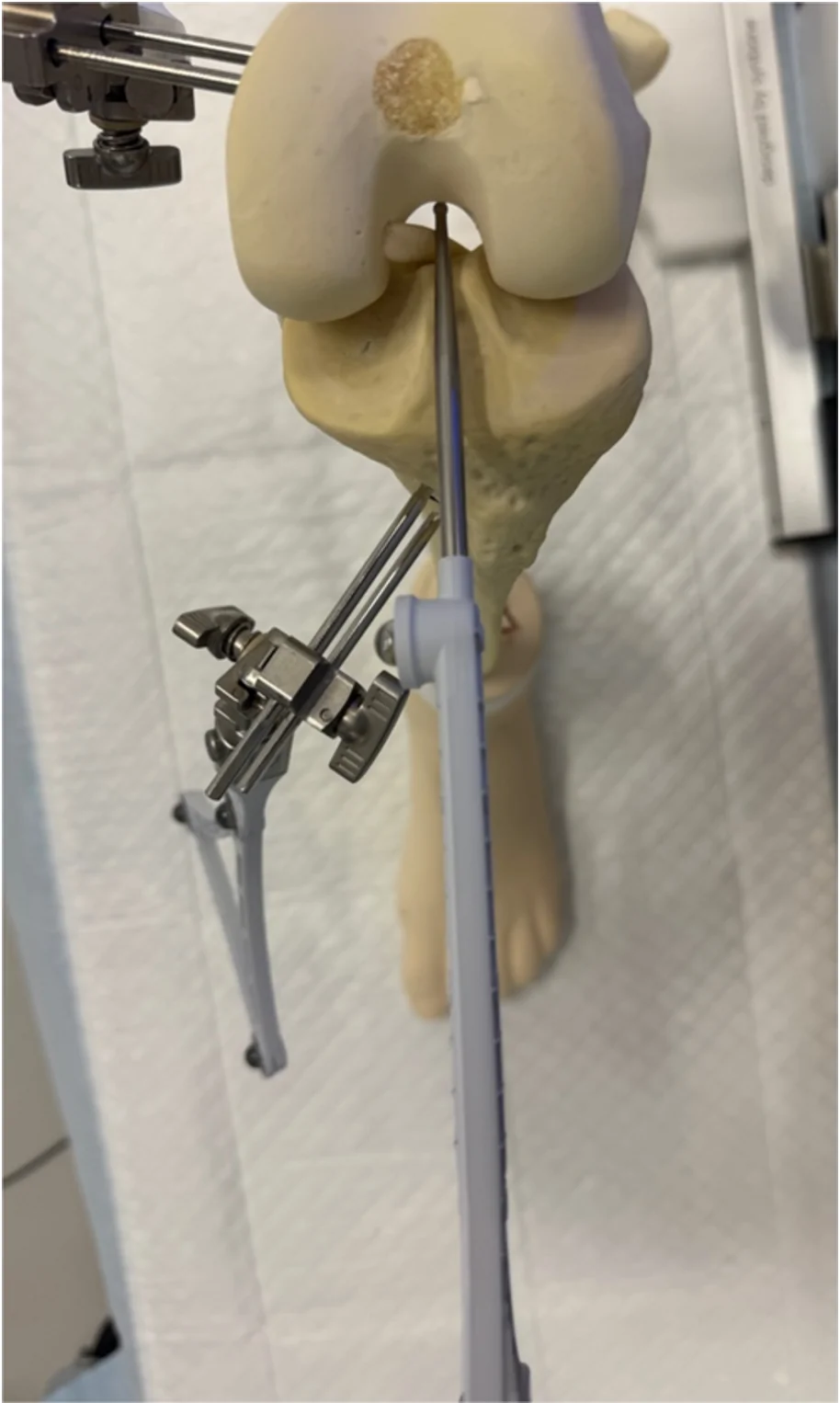
The pointer can also be used as a visual reference to align it according to the Akagi line [12] or the second metatarsal bone, simplifying the definition of the tibia sagittal axis.

In the absence of malrotation of the tibia, the correct definition of the sagittal axis can be simplified by using the pointer orientation as a virtual guide. Viewed from above and aligned according to the Akagi line [4] or the second metatarsal bone, a valid sagittal orientation can be recorded in the majority of cases (Figure 12).

### Femoral rotation

The pointer is used to define Whiteside's line. In the setup screen, the authors recommend using the posterior condyles as the primary reference for defining femoral rotation, because in our experience, it is the more reliable registration process, as 150 data points are collected. Whiteside's line is then used as a secondary reference. The transepicondylar axis can be used for referencing as a third option, though its inter‐ and intra‐observer reliability are low, limiting its value [14].

### Tibia plateau

To correctly define the medial proximal tibial angle (MPTA) of the tibia, it is important to assess cartilage wear on both the medial and lateral tibial plateaus and to factor this into the planning step. If there is a tibial bone defect, the pointer should not be placed within the defect, but rather on the tidemark. If the deepest part of the medial plateau is registered instead, the MPTA will be underestimated, and the resection height of the lateral plateau may be excessive. This is because the software automatically sets the tibial resection at a minimum of 2 mm below the deepest registered point.

**Figure 13:**
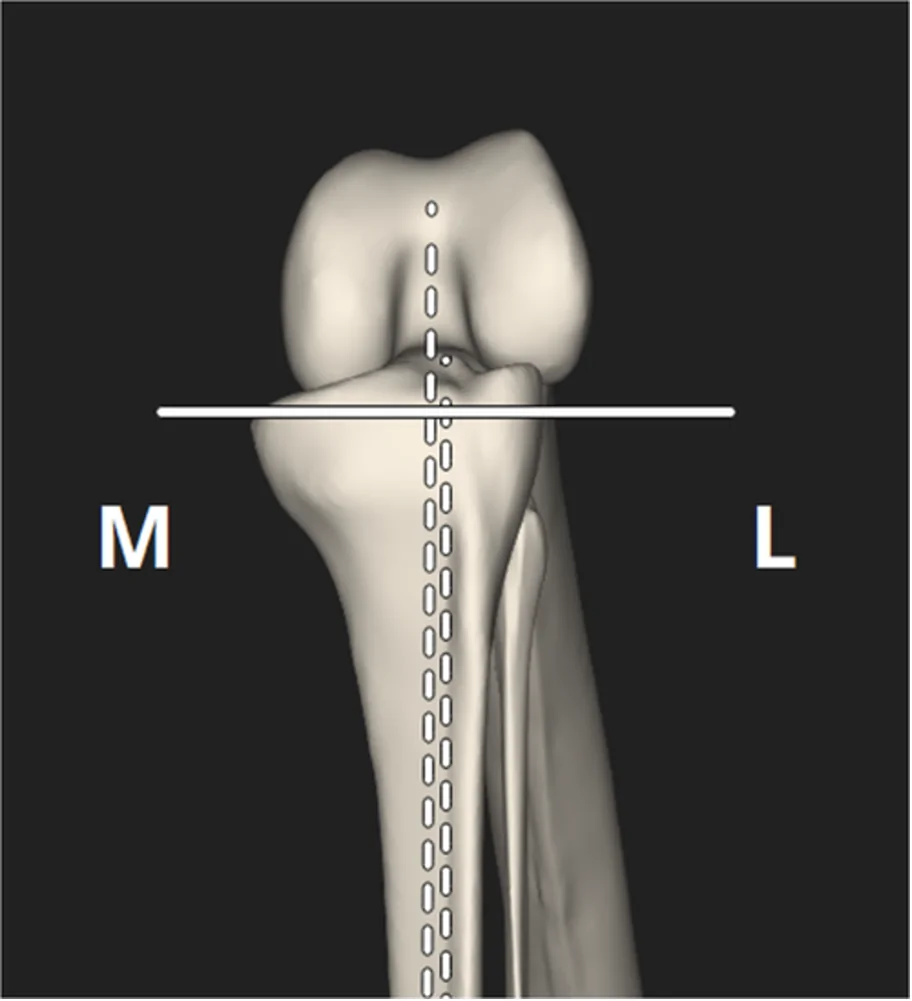
An asymmetric tibia plateau in the 3D visualization model is an indicator of a potential landmarking error of the tibia plateau. 3D, three‐dimensional.

If the lateral plateau is poorly visualized, for example, in cases with a patella baja, the registration may be incorrect. An asymmetric 3D visualization model might indicate this error (Figure 13). In summary, the points of the femoral and tibial sides should be marked at the articulating femorotibial area.

### Distal and posterior femoral condyles

Mapping of the femoral condyles is important for determining the most distal and posterior aspects of the femur on the medial and lateral sides. Incorrect registration can lead to a mismatch in laxity and over‐ or under‐resection of the condyles, which can in turn lead to an inadequate knee balance. Osteophytes should be removed, and marking should be performed at the articulating surface of the femur.

### Anterior femoral cortex

The anterior femoral cortex serves as the reference for femoral sizing. Errors in anterior cortex acquisition may result in femoral over‐ or undersizing. The anterior femoral cortex should not be acquired too far medially, as this might lead to femoral notching. In the authors' experience, registering the anterior cortex 1–2 mm below the apex of the cortex avoids oversizing of the femoral component without risking femoral notching. Cross‐checking with the pointer is recommended. The ‘angel wing’ can be placed on the saw blade when the saw is in position to check for notching or anterior femoral overstuffing. In this option, the 2 mm saw blade thickness also needs to be taken into account. Clearing of hypertrophic capsular tissue and removing osteophytes are recommended before registration, which should be started far proximal in a smooth line, as 7 cm of anterior cortex is required.

If the preceding step of landmarking of the distal femoral condyles has resulted in an airshot, an error message during the anterior femoral cortex landmarking may occur, reporting a failed acquisition (‘acquire anterior cortex line ending more distally’). In this case, the authors recommend double‐checking the mapping of the distal femoral cortices again and re‐doing the registration of these landmarks before reacquiring the anterior femoral cortex.

### Validation of landmarks

After all landmarks have been marked, it is important to check these points. Validation is accurate at 0.2 mm and should only be accepted if displayed in green only. If the landmarks are displayed in red, it is mandatory to reacquire them.

## DYNAMIC GAP MEASUREMENT

In rTKA using the VELYS™ robotic system, intraoperative assessment of knee kinematics is performed following initial mapping of the knee joint as described above. The joint is subsequently subjected to a guided ROM, incorporating standardized varus and valgus stress tests [16]. This process generates an Accubalance graph, providing quantitative laxity values across the flexion‐extension arc for evaluation of soft tissue balance.

It is possible to achieve balanced knee kinematics in the absence of full extension, even in cases presenting with a severe fixed flexion deformity. In this scenario, the authors recommend performing the tibial resection first, with appropriate parameters configured in the robotic system to guide the cut. After the tibial resection, posterior osteophytes and the posterior horns of both menisci can be excised, and if necessary, a posterior capsular release may be performed. A second gap assessment can then be performed, either (a) using a sensor‐tensor or (b) with the trial tibia and insert. In option (a), defined distraction forces are applied. In smaller or very lax knees, this could potentially overstuff the joint and yield falsely large gap values. This is particularly seen in flexion, as this gap is generally looser than the extension gap [5]. In option (b), the tibia base plate with the insert is used instead of the tensor. With this method, dynamic stress testing with similar forces as before must be applied manually. It is important to use the insert with the same height as that shown on the planning screen. In the authors' experience, these interventions facilitate subsequent balancing using the system's balancer tool, ultimately achieving full extension and optimal joint stability.

The Accubalance graph may also reveal hyperlaxity or hyperextension of the knee. In these cases, the authors advise avoiding over‐resection of the tibia to prevent the need for an excessively thick polyethylene insert. We believe that this approach underscores the importance of tailored resection strategies based on intraoperative laxity metrics, aiming to optimize outcomes in rTKA.

## LIMITATIONS

The present manuscript is based on the collective clinical experience of an international panel of eight long‐term VELYS^TM^ users from six countries. When interpreting the information provided in this article, it is important to understand it as a narrative technical guide with no formal consensus methodology, such as a Delphi process. The herein reported recommendations should be regarded as practical guidance rather than evidence‐based safety boundaries. While we agree that this represents a limitation of this present article, the emphasis on accurate landmarking is highly relevant to provide practical guidance for intraoperative recognition of landmarking and gap balance assessment errors during rTKA.

## CONCLUSION

rTKA using the VELYS™ system has gained widespread adoption due to its demonstrated accuracy [2, 3, 11, 12]. However, inaccuracies introduced during the landmarking process, as discussed in this paper, may lead to discrepancies between the intraoperative planning and the postoperative radiographic alignment. Precise acquisition of bony landmarks and meticulous intraoperative verification are essential to obtaining the best possible result. This article aims to support early adopters of the VELYS™ system by providing practical guidance based on the collective clinical experience of an international panel of high‐volume users for intraoperative assessment of correct registration and prevention of alignment inaccuracies. Cadaveric studies are warranted to better understand the exact influence of each technical error on system precision.

## AUTHOR CONTRIBUTIONS

**All authors**: Substantial contributions to the conception or design of the work; or the acquisition, analysis or interpretation of data for the work; drafting the work or reviewing it critically for important intellectual content; final approval of the version to be published; and agreement to be accountable for all aspects of the work in ensuring that questions related to the accuracy or integrity of any part of the work are appropriately investigated and resolved.

## CONFLICT OF INTEREST STATEMENT

There are no conflicts of interest directly related to the content of this article. The authors report receiving royalties from Enovis and Lima; honoraria for presentations from Johnson & Johnson (DePuy Synthes), Medacta and Ethicon; consulting fees from Johnson & Johnson (DePuy Synthes), Medacta, Ethicon, Symbios, Smith & Nephew and Hectec; stock or stock options in Revel AI, VISIE and ROMTECH; and research support from Johnson & Johnson (DePuy Synthes), Medacta and Zimmer Biomet. The authors also serve on the editorial or governing boards of *Bone & Joint 360*, *Knee Surgery, Sports Traumatology, Arthroscopy* and *Journal of Arthroplasty* and hold leadership or membership roles in the Israeli Hip & Knee Society, American Association of Hip and Knee Surgeons, the German Knee Society, European Society of Sports Traumatology, Knee Surgery and Arthroscopy, European Society of Sports Traumatology, Knee Surgery and Arthroscopy and the Belgian Knee Society.

## ETHICS STATEMENT

The authors have nothing to report.

## Data Availability

The authors have nothing to report.
